# Plasma Metabolomics Analysis of Polyvinyl Chloride Workers Identifies Altered Processes and Candidate Biomarkers for Hepatic Hemangiosarcoma and Its Development

**DOI:** 10.3390/ijms22105093

**Published:** 2021-05-11

**Authors:** John J. Guardiola, Josiah E. Hardesty, Juliane I. Beier, Russell A. Prough, Craig J. McClain, Matthew C. Cave

**Affiliations:** 1 Department of Medicine, University of Louisville, Louisville, KY 40202, USA; joguar@iu.edu (J.J.G.); josiah.hardesty@louisville.edu (J.E.H.); craig.mcclain@louisville.edu (C.J.M.); 2 Hepatology and Nutrition, University of Louisville Division of Gastroenterology, Louisville, KY 40202, USA; 3 Division of Gastroenterology, Hepatology and Nutrition, Department of Medicine, University of Pittsburgh, Pittsburgh, PA 15213, USA; jibeier@pitt.edu; 4 Department of Environmental and Occupational Health, University of Pittsburgh, Pittsburgh, PA 15213, USA; 5 University of Pittsburgh Liver Research Center (PLRC), Department of Medicine, University of Pittsburgh, Pittsburgh, PA 15213, USA; 6 Department of Biochemistry and Molecular Genetics, University of Louisville, Louisville, KY 40202, USA; russell.prough@louisville.edu; 7 Department of Pharmacology and Toxicology, University of Louisville, Louisville, KY 40202, USA; 8 The Robley Rex Veterans Affairs Medical Center, Louisville, KY 40206, USA; 9 The UofL Health—Jewish Hospital Trager Transplant Center, Louisville, KY 40202, USA; 10 The University of Louisville Superfund Research Center, Department of Medicine, University of Louisville, Louisville, KY 40202, USA

**Keywords:** angiosarcoma, vinyl chloride, PVC, liver cancer

## Abstract

Background: High-level occupational vinyl chloride (VC) exposures have been associated with hepatic hemangiosarcoma, which typically develops following a long latency period. Although VC is genotoxic, a more comprehensive mode of action has not been determined and diagnostic biomarkers have not been established. The purpose of this study is to address these knowledge gaps through plasma metabolomics. Methods: Plasma samples from polyvinyl chloride polymerization workers who developed hemangiosarcoma (cases, *n* = 15) and VC exposure-matched controls (*n* = 17) underwent metabolomic analysis. Random forest and bioinformatic analyses were performed. Results: Cases and controls had similar demographics and routine liver biochemistries. Mass spectroscopy identified 606 known metabolites. Random forest analysis had an 82% predictive accuracy for group classification. 60 metabolites were significantly increased and 44 were decreased vs. controls. Taurocholate, bradykinin and fibrin degradation product 2 were up-regulated by greater than 80-fold. The naturally occurring anti-angiogenic phenol, 4-hydroxybenzyl alcohol, was down-regulated 5-fold. Top affected ontologies involved: (i) metabolism of bile acids, taurine, cholesterol, fatty acids and amino acids; (ii) inflammation and oxidative stress; and (iii) nicotinic cholinergic signaling. Conclusions: The plasma metabolome was differentially regulated in polyvinyl chloride workers who developed hepatic hemangiosarcoma. Ontologies potentially involved in hemangiosarcoma pathogenesis and candidate biomarkers were identified.

## 1. Introduction

Vinyl chloride monomer (VCM or VC, CAS ID# 75-01-4) is a gaseous haloalkene that is polymerized to form the ubiquitous plastic polyvinyl chloride (PVC). While occupational and environmental VC exposures have been associated with hepatotoxicity and chemical carcinogenesis, PVC is believed to be relatively non-toxic. Both occupational and environmental VCM exposures occur and VC ranks #4 on the Agency for Toxic Substances & Diseases Registry 2019 Substance Priority List. VCM exposures have been associated with liver cancers including hemangiosarcoma (previously called angiosarcoma of the liver, ASL) and hepatocellular carcinoma (HCC).

Hepatic hemangiosarcoma is a malignant vascular tumor of sinusoidal endothelial cells and it is rare in humans. Although sporadic cases occur, human cases have typically been associated with exposures to VCM, thorotrast, or arsenic. In contrast, sporadic ASLs occur at a higher frequency in other mammalian species [1]. In these species, ASLs are also associated with exposures to additional environmental chemicals and even certain medications. VCM’s mode of action (MOA) in ASL is believed to be due to the generation of the reactive chloroethylene oxide intermediate, which then adducts DNA resulting in genotoxicity [2]. DNA mutations in the K-ras oncogene and the P53 tumor suppressor gene have been reported in some, but not all, cases of human VC-induced ASL [2]. Nongenotoxic proliferative mechanisms in hemangiosarcoma leading to dysregulated angiogenesis and/or erythropoiesis have been proposed [1]. The present manuscript aims to elucidate further the biology of VCM-related human ASL by performing pathway analysis on differentially regulated plasma metabolites.

The identification of hepatic hemangiosarcoma in PVC production workers at a polymerization plant in Louisville, KY in 1974 was a sentinel event in occupational health [3,4]. These workers were typically ‘poly cleaners’ and they had prolonged high-level VCM exposures while manually cleaning PVC polymerization reactors at the Rubbertown chemical manufacturing complex. This recognition prompted the introduction of 1975′s Vinyl Chloride Standard, which greatly reduced occupational VCM exposures and mandated medical surveillance in workers. Hepatic hemangiosarcoma was subsequently recognized in VC workers worldwide prompting the creation of a global case registry. To date, 28 confirmed ASL cases have occurred in workers from the Louisville facility alone; while the most recent summary report of 35 US facilities through 2013 identified 63 ASLs, 32 HCCs and 36 unspecified liver cancers [5]. This recent report was consistent with the Louisville experience in that ASLs occurred following a long latency period (median 36 years) and only in workers who had high estimated cumulative VCM exposures [>1000 parts per million (PPM)-years]. Compared with workers with <1021 ppm-years, workers exposed to ≥10,551 ppm-years had an ASL hazard ratio of 73.6 [5].

At the Louisville plant, the Vinyl Chloride Standard appears to have prevented the development of ASL in workers hired after its implementation; however, due to ASL’s long latency period, VC workers with remote high-level exposures are still at risk. In fact, such a case recently occurred in a retiree from the Louisville plant, with another recent case occurring in Italy [6]. Liver cancer is associated with occupational exposures to VC [7]. Liver cancer from VC exposures has gained attention clinically leading to the publication of Clinical Practice Guidelines for Occupational Liver Diseases [8]. ASL is a highly aggressive tumor and is typically diagnosed late when the cancer is already at an advanced stage. Thus, the median survival was only 5 months in a recent treatment series [9]. Liver transplantation is contraindicated due to a high recurrence rate. When the tumor is diagnosed early, optimal management involves local resection with or without adjuvant therapy. Prolonged survival has occurred following this approach.

Because (i) at risk workers (most likely now retirees) can be identified by their occupational exposure records; (ii) ASL has a long latency period and cases still occur; and (iii) an effective therapy is available for early disease, ASL surveillance still appears warranted. The optimal screening strategy is unknown, but like hepatocellular carcinoma, it could potentially involve the combination of serial imaging and blood-based biomarkers. The circulating biomarkers of VCM-related ASL are currently unknown. Even though serial routine liver chemistry panels are mandated by the Vinyl Chloride Standard, these biomarkers were typically normal in the ASL cases from the Louisville plant [10]. The present manuscript, in part, seeks to discover new blood-based candidate biomarkers for human ASL through a plasma metabolomics approach to address this unmet medical need.

The University of Louisville’s unique occupational surveillance biorepository founded by the late Dr. Carlo Tamburro will be utilized to perform this work. This biorepository was established in the 1970′s through an innovative partnership between the University, Rubbertown’s chemical producers and the National Cancer Institute. As a component of medical surveillance, data and biological specimens were archived at least annually for future research. The Cave laboratory has utilized this resource to identify biopsy-proven toxicant-associated steatohepatitis (TASH) occurring in PVC workers [11]. Subsequently, the laboratory demonstrated that PVC workers had an altered plasma metabolome and liver processes/pathways compared to unexposed controls [12]. Building on the latter study, we hypothesize that metabolomics analysis of archived plasma samples from similarly exposed PVC workers with and without ASL will yield new candidate biomarkers and elucidate new pathways/processes for vinyl chloride-induced human hemangiosarcoma. This work is relevant to at-risk chemical workers and to investigators studying chemical carcinogenesis.

## 2. Results

### 2.1. Demographics, Chemical Exposure and Clinical Chemistries of Hemangiosarcoma and Control Cohorts

All workers were Caucasian males. The mean age (50.5 ± 6.3 vs. 56.2 ± 10.9 years), duration of employment at time of sampling (25.5 ± 5.7 vs. 31.9 ± 12.0 years), body mass index (27.1 ± 3.4 vs. 25.3 ± 2.1 kg/m^2^), CERM (1156 ± 420.4 vs. 1266 ± 541.2), albumin (4.6 ± 0.2 vs. 4.4 ± 0.2 mg/dL), total bilirubin (0.6 ± 0.2 vs. 0.6 ± 0.2 mg/dL), alkaline phosphatase (70.5 ± 15.0 vs. 68.3 ± 36.2 U/L), AST (24.1 ± 15.6 vs. 27.8 ± 12.1 U/L), ALT (24.9 ± 18.4 vs. 19.8 ± 8.3 U/L), triglycerides (153 ± 49.6 vs. 201 ± 101.6 mg/dL), blood glucose (101 ± 17.3 vs. 105 ± 16.1 mg/dL) and cholesterol (213 ± 32.6 vs. 217 ± 32.5 mg/dL) of Control versus Hemangiosarcoma group were not significantly different between groups.

The age of samples of Hemangiosarcoma group was significantly less (*p* < 0.05) than the age of samples of Control group (27.5 ± 11.8 vs. 35.0 ± 5.1 years). This was secondary to methodology of the study—some samples were drawn during follow-up period as patients were diagnosed with hemangiosarcoma. (Table 1 and Table 2).

### 2.2. Metabolites Altered in the Development of vc-Mediated Hemangiosarcoma

**Figure 1:**
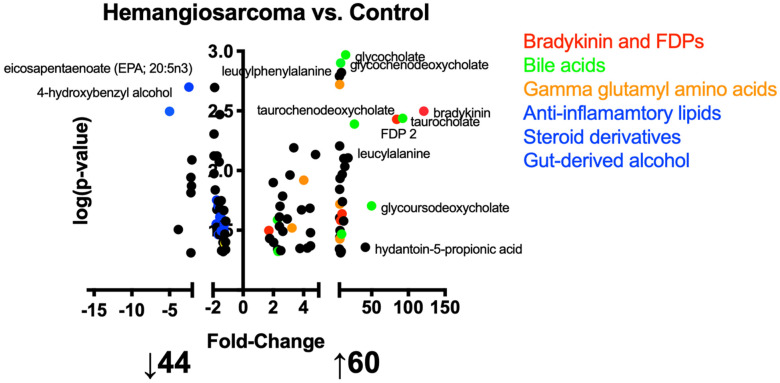
Metabolites altered in the development of VC-mediated hemangiosarcoma. Volcano plots of metabolite data significantly different when comparing Hemangiosarcoma vs. Control. Data are plotted as fold-change vs. -log(*p*-value). Metabolomic datasets were statistical compared by a Welch’s *t*-test with a *p* < 0.05 being considered significant.

A metabolomics approach was used to identify metabolic changes in the development of VC-induced hemangiosarcoma. A total of 1123 metabolites were identified of which 606 metabolites were assigned identities. Figure 1 is a volcano plot of identified metabolites that demonstrates fold change, specifically that 44 metabolites were decreased when comparing Hemangiosarcoma vs. Control and 60 were increased. Metabolites in red include bradykinin, C3 and fibrin degradation products (FDPs). Metabolites in green are bile acids, orange are gamma glutamylated amino acids and yellow are anti-inflammatory lipids, steroid derivatives and a gut-derived phenol. Supplementary Materials Tables S1–S3 list identified metabolites that were unchanged, increased and decreased when comparing Hemangiosarcoma vs. Control.

### 2.3. Ontologies Implicated in the Development of VC-Mediated Hemangiosarcoma

Limited pathways and processes have been implicated in the development of VC-mediated hemangiosarcoma and thus ontology analyses were conducted to interpret significant metabolomic data. Table 3 demonstrates multiple ontologies enriched in Hemangiosarcoma involving both bile acid and cholesterol metabolism, specifically, bile acid biosynthesis, taurine and hypotaurine metabolism, cholesterol metabolism, cholesterol and bile homeostasis. Additionally, ontologies related to inflammation were enriched, including upregulation of IL-8 expression in colorectal cancer and oxidative phosphorylation. Table 4 demonstrates ontologies diminished in Hemangiosarcoma including amino acid synthesis and cholesterol and steroid metabolism.

### 2.4. Serum Inflammatory Proteins Bradykinin and C3 Are Elevated while Anti-Inflammatory Lipids Are Diminished in VC-Mediated Hemangiosarcoma

**Figure 2:**
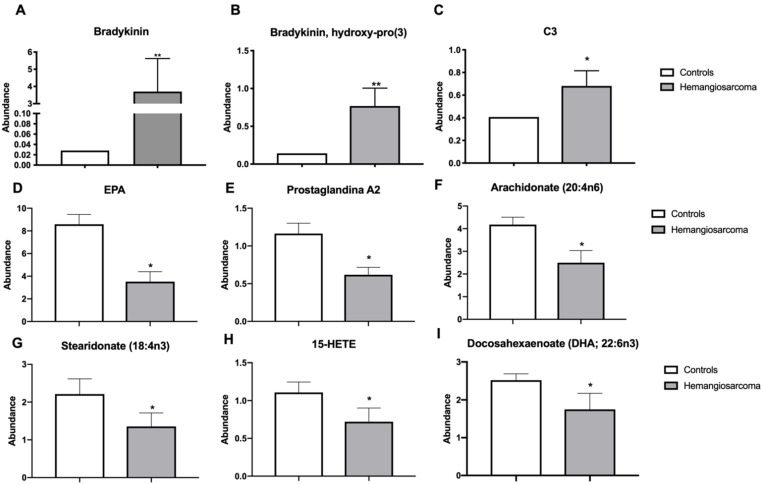
Serum inflammatory proteins bradykinin and C3 are elevated while anti-inflammatory lipids are diminished in VC-mediated hemangiosarcoma. Serum bradykinin (**A**), hydroxylated bradykinin (**B**), C3 (**C**), EPA (**D**), prostaglandin A2 (**E**), arachidonate (20:4n6) (**F**), stearidonate (18:4n3) (**G**), 15-HETE (**H**) and DHA (22:6n3) (**I**) are plotted for Control and Hemangiosarcoma groups. Data are represented as mean ± SEM. * denotes significance with *p* < 0.05, ** *p* < 0.01.

Bradykinin is a peptide that is involved in inflammation and acts as a vasodilator that lowers blood pressure [13]. Bradykinin was upregulated in Hemangiosarcoma vs. Control (121-fold, *p* = 0.0032). The metabolite bradykinin hydroxyproline was upregulated in Hemangiosarcoma group vs. Control (5.7-fold, *p* = 0.0029). Complement component 3 (C3) is a hepatic derived protein involved in innate immune responses and inflammation [14]. C3 was elevated in Hemangiosarcoma vs. Control (1.71-fold, *p* = 0.0261) (Figure 2A–C). Anti-inflammatory lipid species act locally to resolve inflammation and in their absence chronic inflammation can occur progressing to neoplasms [15]. The following lipids were reduced in Hemangiosarcoma: eicosapentaenoic acid (EPA) (2.38-fold, *p* = 0.002), prostaglandin A2 (1.92-fold, *p* = 0.002), arachidonate (1.7-fold, *p* = 0.0198), stearidonate (1.59-fold, *p* = 0.0344), 15-HETE (1.49-fold, *p* = 0.0245) and docosahexaenoic acid (DHA) (1.52-fold, *p* = 0.0321) (Figure 2D–I).

### 2.5. Serum Bile Acids Are Increased in VC-Mediated Hemangiosarcoma

**Figure 3:**
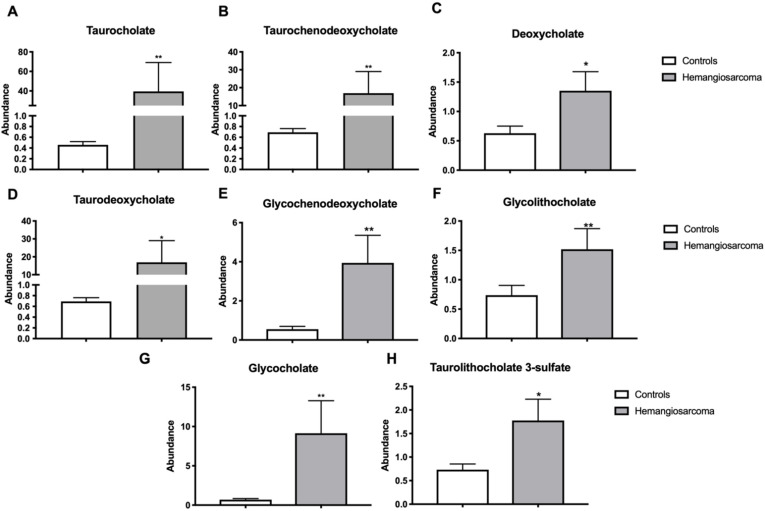
Serum bile acids are increased in VC-mediated hemangiosarcoma. Serum glycocholate (**A**), taurocholate (**B**), taurochenodeoxycholate (**C**), deoxycholate (**D**), taurodeoxycholate (**E**), glycochenodeoxycholate (**F**), glycolithocholate sulfate (**G**) and taurolithocholate 3-sulfate (**H**) are plotted for Control and Hemangiosarcoma groups. Data are represented as mean ± SEM. * denotes significance with *p* < 0.05, ** *p* < 0.01.

Bile acid synthesis from cholesterol and sequestering into the gut are two critical functions of the liver in lipid metabolism [16]. The bile acids glycocholate (15-fold, *p* = 0.0011), taurocholate (92-fold, *p* = 0.0036), taurochenodeoxycholate (27-fold, *p* = 0.0041), deoxycholate (2.3-fold, *p* = 0.0476), taurodeoxycholate (9-fold, *p* = 0.0341), glycochenodeoxycholate (7.8-fold, *p* = 0.0013), glycolithocholate sulfate (2.3-fold, *p* = 0.0259) and taurolithocholate 3-sulfate (2.6-fold, *p* = 0.0323) were all upregulated in Hemangiosarcoma (Figure 3A–H).

### 2.6. Serum Gamma-Glutamyl Amino Acids Are Increased in VC-Mediated Hemangiosarcoma

**Figure 4:**
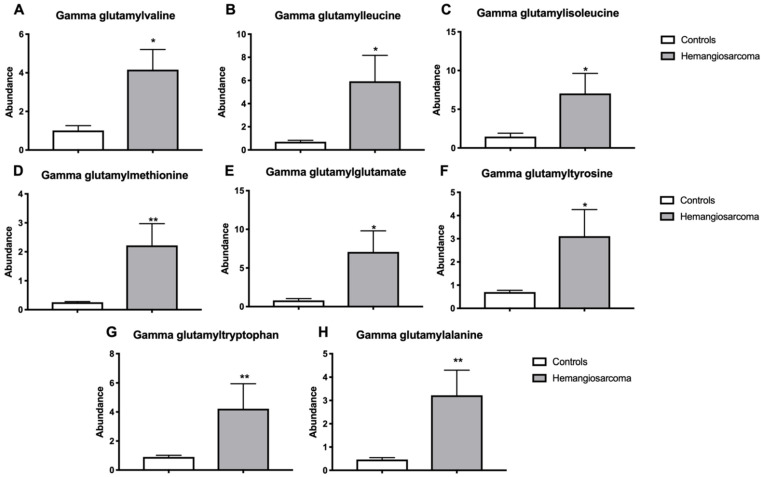
Serum gamma-glutamyl amino acids are increased in VC-mediated hemangiosarcoma. Serum gamma-glutamylvaline (**A**), gamma-glutamylleucine (**B**), gammaglutamylisoleucine (**C**), gamma-glutamylmethionine (**D**), gamma-glutamylglutamate (**E**), gamma-glutamyltyrosine (**F**), gamma-glutamyltryptophan (**G**) and gamma-glutamylalanine (**H**) are plotted for control group, Control and Hemangiosarcoma groups. Data are represented as mean ± SEM. * denotes significance with *p* < 0.05, ** *p* < 0.01.

Gamma-glutamyl transferase is an enzyme that catalyzes the transfer of gamma-glutamyl groups from glutathione to amino acids and is indicative of oxidative stress and its expression is often upregulated in cancer [17]. Gamma-glutamylvaline (3.2-fold, *p* = 0.0303), gamma-glutamylleucine (6.7-fold, *p* = 0.0374), gammaglutamylisoleucine (4.5-fold, *p* = 0.0427), gamma-glutamylmethionine (5.7-fold, *p* = 0.0016), gamma-glutamylglutamate (6.6-fold, *p* = 0.0191), gamma-glutamyltyrosine (4.0-fold, *p* = 0.012), gamma-glutamyltryptophan (4.8-fold, *p* = 0.0073), gamma-glutamylalanine (6.3-fold, *p* = 0.0019) were all upregulated in Hemangiosarcoma workers when compared with Control workers (Figure 4A–H).

### 2.7. Fibrin Degradation Products Are Elevated in VC-Mediated Hemangiosarcoma

**Figure 5:**
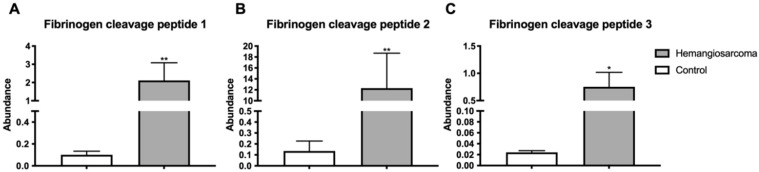
Fibrin degradation products are elevated in VC-mediated hemangiosarcoma. Serum fibrinogen peptides Fibrin Degradation Product 1 (**A**), Fibrin Degradation Product 2 (**B**), Fibrin Degradation Product 3 (**C**) are plotted for Control and Hemangiosarcoma groups. Data are represented as mean ± SEM. * denotes significance with *p* < 0.05, ** *p* < 0.01.

Fibrin is a protein involved in blood clot formation. Fibrin degradation products are components of blood that are produced from clot breakdown [18]. Three fibrin cleavage peptides were identified and all three were significantly elevated in Hemangiosarcoma when compared to Control workers—Fibrin Degradation Product 1 (10-fold, *p* = 0.0037), Fibrin Degradation Product 2 (84-fold, *p* = 0.0085), Fibrin Degradation Product 3 (5.8-fold, *p* = 0.023) (Figure 5A–C).

### 2.8. Steroid Hormones, Steroid Hormone Metabolites and Gut-Derived Phenol Gastrodigenin Are Diminished in VC-Mediated Hemangiosarcoma

**Figure 6:**
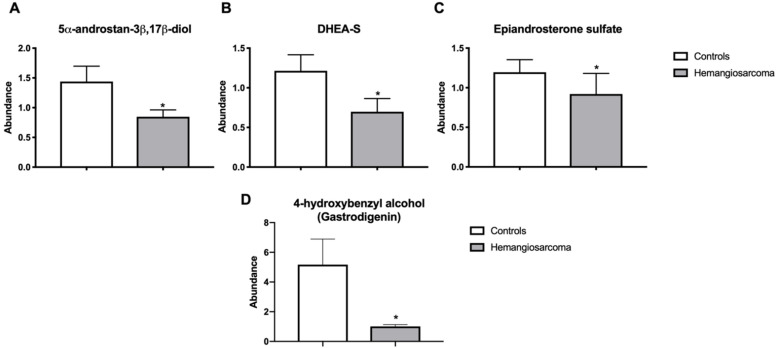
Steroid hormones, steroid hormone metabolites and gut-derived phenol gastrodigenin are diminished in VC-mediated hemangiosarcoma. Serum 5alpha-androstan-3-beta,17-beta-diol disulfate (**A**), Dehydroisoandrosterone (DHEA) sulfate (**B**), Epiandrosterone (**C**) and gastrodigenin (**D**) are plotted for Control and Hemangiosarcoma groups. Data are represented as mean ± SEM. * denotes significance with *p* < 0.05.

The liver is instrumental in the metabolism of many steroid hormones, including testosterone and DHEA [19]. 5alpha-androstan-3-beta,17-beta-diol disulfate, an estrogen receptor-beta agonist, was diminished (1.8-fold, *p* = 0.028) in Hemangiosarcoma relative to Control. Dehydroepiandrosterone (DHEA) sulfate was decreased (1.8-fold, *p* = 0.0177) in hemangiosarcoma relative to Control. Epiandrosterone was diminished (1.3-fold, *p* = 0.032) in hemangiosarcoma (Figure 6A–C). The gut-derived phenol gastrodigenin was decreased (5-fold, *p* = 0.0032) in hemangiosarcoma (Figure 6D).

### 2.9. Random Forest Analysis Demonstrates That Metabolites from the Hemangiosarcoma and Control Are Statistically Distinct

**Figure 7:**
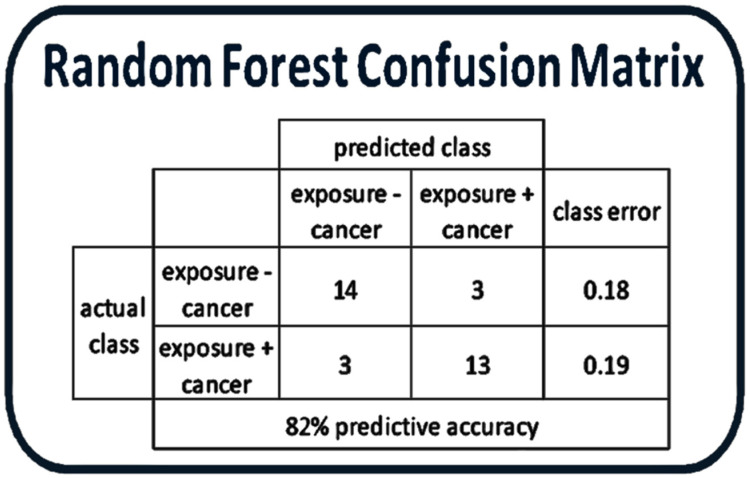
Random forest confusion matrix and biochemical importance plot. Confusion matrix for Hemangiosarcoma and Control.

Random forest analysis was performed between Hemangiosarcoma and Control groups to determine if the metabolic data could successfully separate these two groups. Fourteen of seventeen (82%) VC subjects could be separated from Hemangiosarcoma cohort with a class error of 0.18 (Figure 7). The F1 score for this classification model was 0.8125.

## 3. Discussion

In metabolomic studies of sarcomas, others have used pooled groups of similar sarcomas [20,21]. Our study is novel in that our study group had the same sarcoma malignancy: hepatic hemangiosarcoma. Additionally, it builds upon our previous study demonstrating the alterations VC has on the plasma metabolome when compared to healthy unexposed controls [12]. Our study also elucidates potential mechanisms and markers for hepatic hemangiosarcoma development following vinyl chloride exposure.

Bradykinin (BK) stimulates angiogenesis via activation of two G-protein coupled receptor subtypes: B1 and B2. B1 is inducible and is upregulated via tissue damage, ischemia, or inflammation while B2 is constitutively expressed [22]. Bradykinin has been demonstrated to increase angiogenesis via B2 receptor activation and has also demonstrated upregulation of vascular endothelial growth factor secondarily leading to angiogenesis [23]. Additionally, BK has been shown to act through Akt [24], which we have previously demonstrated is likely altered in vinyl chloride exposure [12,25]. Icatibant is a B2 receptor inhibitor primarily prescribed for treatment of angioedema [26], but based on our data, it could hold potential for prevention or treatment of vinyl chloride induced hemangiosarcoma. There are three subtypes of the VEGF receptor and each has been demonstrated to be upregulated in angiosarcoma [27,28]. A metabolite of bradykinin, hydroxyprolyl3 bradykinin has been measured previously in ascites fluid of multiple cancers including gastric and ovarian [29,30]. Our findings support previous reports of bradykinin and hydroxypro3 bradykinin as possible biomarkers for hemangiosarcoma and the upregulation of bradykinin and its metabolite as potential mechanisms of vinyl chloride-induced hepatic hemangiosarcoma. VEGF receptor and PDGF receptor inhibitors have shown potential for the treatment of angiosarcomas [31,32] and could hold potential for treating vinyl chloride associated hemangiosarcoma as well.

C3 is an innate immune response protein that is released from the liver during inflammation [14] and was elevated in hemangiosarcoma. Tissue inflammation in the development of sarcoma is well-characterized [33] and C3 could be a biomarker for hemangiosarcoma risk in chemical workers with high cumulative VC exposures.

All necessary components of the fibrinolytic pathway have been identified on the surface of tumor cells, including urokinase-type (u-PA), tissue-type plasminogen activator (t-PA), plasminogen activator inhibitor-1 (PAI-1) and plasminogen activator inhibitor-2 (PAI-2) [34]. Tumor cells may activate the fibrinolytic system by releasing coagulation factors or plasminogen activators. Thrombin acts as a growth factor for tumor cells and facilitates tumor angiogenesis leading to fibrin formation [35]. PA generates plasmin that promotes invasion and migration of tumor cells into the circulation [36]. Plasmin activity leads to fibrin degradation products. Fibrin degradation products (FDPs) have been described as angiogenic [37]. Previous studies have shown fibrin degradation products are elevated in lung, ovarian and a variety of gastrointestinal cancers and have shown potential to be used as biomarkers [38,39,40,41]. VC has been shown to activate the coagulation system and to increase fibrin deposition in hepatic tissue [42]. Our study is the first to identify elevated circulating FDPs in humans that developed hemanigosarcomas [43].

Bile acids are metabolized from cholesterol and secreted by the liver. Alterations of bile acid serum levels are therefore considered a liver-specific marker. Bile acids have been identified as clinically usable biomarkers of VC-induced hepatic injury in exposed asymptomatic workers [44]. In our study, several bile salts were elevated in the Hemangiosarcoma group and multiple ontologies involving bile acid metabolism were enriched in the pathway analysis while steroid metabolism was downregulated. Taurocholic acid and glycocholate have been identified as biomarkers of cirrhosis [45] and of human hepatocellular carcinoma [46]. Taurocholate and other conjugated bile acids have also been shown to promote cholangiocarcinoma cell-invasive growth [47] and glycoursodeoxycholate has been shown to be elevated in gallbladder cancer [48]. It has been shown that taurocholic acid caused duodenal angiosarcoma in rats [49]; however, the potential mechanistic role of bile acids in hepatic angiosarcoma is unclear. VC exposure in humans increases metabolites that interact with Akt signaling and that Akt activation was enhanced after VC metabolite exposure in mice [12,25]. Bile acids increase endothelial nitric oxide synthase (eNOS) in sinusoidal endothelial cells. Taurocholate and other bile acids have been shown to activate both Akt and ERK1/2 [50]. The Akt-eNOS pathway is required for initiation and maintenance of tumor growth [51] and angiosarcoma has been demonstrated to be positive for eNOS [52]. Our study suggests specific bile acids, notably taurine conjugates especially taurocholate, may be useful as biomarkers and that alterations of bile acids likely play an important role in VC-induced toxicity and development of VC-related hemangiosarcoma.

Gamma-glutamylation increases solubility of amino acids and peptides, stabilizes these compounds in the bloodstream and may alter signaling processes [53]. Elevated gamma-glutamyl transferase (GGT) activity is a marker of not only liver damage but also of oxidative stress [54]. Oxidative stress was one of the ontologies identified based on the metabolites increased in the VC-exposed workers who developed hemangiosarcoma. This would suggest that oxidative stress from GGT activity is a contributing factor in VC-mediated hemangiosarcoma. Gamma-glutamyl dipeptides have been identified as novel liver biomarkers for differentiation between different forms of liver disease [55] and identified as markers of response to treatment of liver disease [56]. Our results demonstrated an increase in multiple γ-glutamyl amino acids in the Hemangiosarcoma group. Gamma-glutamyl amino acids have been identified as potential biomarkers in multiple cancers [57,58]. GGT activity has been shown to be increased in angiosarcoma [59,60] supporting the hypothesis that gamma-glutamyl amino acids could be valid biomarkers for VC-induced hemangiosarcoma.

Gastrodigenin, also known as 4-hydroxybenzyl alcohol, is a metabolite produced during the biosynthesis of thiamine by *E. coli* [61] and was found to be significantly decreased in Hemangiosarcoma. Gastrodigenin has been found to have anti-angiogenic and anti-tumor effects [62]. This may be due in part to upregulation of Nrf2 and other antioxidant pathways [63]. We have previously demonstrated vinyl chloride metabolites directly damage mitochondria [25]. Gastrodin, the pro-metabolite of gastrodigenin, was shown to act via nrf2 induction to confer protection to mitochondria and cells against pro-oxidant agents [64]. Gastrodigenin has been shown to down-regulate expression of VEGF and MMP9, both important in angiogenesis. Additionally, it was demonstrated to inhibit endothelial cell migration [65]. These findings, along with the bile acid findings, may point to a role of alteration of the gut microbiome in the VC-mediated development of hemangiosarcoma.

Previously, we showed VC-exposed workers had significant increases of oxidized lipids, most notably 15-HETE (615-fold increase), compared to unexposed controls [12]. 15-HETE, an arachidonic acid derived metabolite of 15-lipoxygenase (15-LOX), is an endogenous ligand for PPAR-γ and has been thought to play a role in multiple human cancers. EPA and DHA, both n-3 polyunsaturated fatty acids, have both shown dose-dependent induction of apoptosis in multiple human solid and hematological tumors [66] and in some instances been shown to act via PPAR-γ to induce apoptosis [67,68]. In a study comparing gene expression of normal epithelial cell lines and epithelial cancer cell lines, 15-LOX expression was high and PPAR-gamma expression was low in normal epithelial cells while the inverse was true of the cancer cell lines [69]. 15-HETE has been demonstrated to inhibit cell growth and induce apoptosis via PPAR-gamma-mediated pathways in several human cancers [70,71,72]. Similarly, the pro-inflammatory IL-8 and colorectal cancer ontology was overrepresented in VC workers who developed hemangiosarcoma. Alterations in inflammatory pathways play a role in colorectal carcinogenesis [73]. Our results show that the pro-inflammatory state may contribute to VC-mediated hemangiosarcoma. 15-HETE and other oxidized lipids likely play a key role in the development of VC-mediated hemangiosarcoma. It is not clear whether the inability to generate sufficient anti-inflammatory lipids may lead to conditions predisposing to hemangiosarcoma or the development of hemangiosarcoma may lead to decreased production of these lipids.

Many of the metabolomic changes in VC-exposed workers who developed hemangiosarcoma are related to inflammatory and oxidative stress related processes with reductions in pro-resolving lipid mediators. The random forest analysis further demonstrates that the VC-exposed workers without hemangiosarcoma can be differentiated from the VC-exposed workers who developed hemangiosarcoma with 82% accuracy based solely on plasma metabolomic changes. This adds support to the metabolites identified in this study, serving as biomarkers for VC-mediated hemangiosarcoma.

Limitations of our study include differing ages of samples given that samples from some of the workers were taken significantly later than some of the exposed workers without hemangiosarcoma. This was due to the nature of the surveillance program and once workers were diagnosed with hemangiosarcoma plasma samples were taken at that time. Plasma samples were frozen for varying degrees of time, but samples were processed for metabolomics studies at the same time. Another limitation of this study is the relatively small sample size. This is due to the limited number of VC-exposed individuals enrolled in the surveillance program and the rare occurrence of hepatic hemangiosarcoma. While the sample is size is low, this is one of the first studies to use an unbiased approach to identify metabolites that may distinguish workers at increased risk for VC-mediated hemangiosarcoma.

## 4. Materials and Methods

### 4.1. Subjects

De-identified data and plasma samples were obtained for highly exposed vinyl chloride workers without cancer (*n* = 17) (henceforth Control) and highly exposed vinyl chloride workers who developed hepatic hemangiosarcoma during follow-up (*n* = 15) (henceforth Hemangiosarcoma) from the University of Louisville Occupational Toxicology Specimen Bank. Samples were stored at −80 °C in polypropylene microfuge tubes. They were thawed, aliquoted and sent on dry ice to Metabolon for analysis. There were no other known freeze-thaw cycles. These workers were employed at a single B.F. Goodrich plant in Louisville, KY and were selected (inclusion criteria) based on exceptionally high cumulative VC exposures prior to implementation of the Occupational Safety and Health Administration (OSHA) Vinyl Chloride Standard in 1975; however, workers were also simultaneously exposed to a variety of other chemicals in addition to VC [11]. These 17 workers never developed hepatic hemangiosarcoma during a prolonged follow-up period, now approximately 40 years in duration. Their toxicant associated steatohepatitis (TASH) status was unknown. Cumulative VC exposures were quantified by the previously described cumulative exposure rank month (CERM) [11,74]. Samples were selected based on their similar CERMS. Study designed to match key demographic and exposure variables with the only difference between groups being development of hepatic hemangiosarcoma. The human subjects protocol was approved by the University of Louisville Institutional Review Board (IRB Number 94.0261 approved 14 January 2020) and informed consent was obtained.

### 4.2. Metabolomics

Metabolon, Inc. (Durham, NC, USA) performed gas chromatography/mass-spectrometry (GC/MS) (Thermo-Finnigan Trace DSQ fast-scanning single-quadruple mass spectrometer) and liquid chromatography-tandem mass spectrometry LC/MS/MS (Waters ACQUITY UPLC, Thermo-Finnigan LTQ mass spectrometer) following metabolite extraction. Software was used to match ions to a library of standards for metabolite identification and for metabolite quantitation by peak area integration. Identified metabolite abundances were scaled to achieve a median equal to 1 and missing values were imputed with the minimum value for the respective metabolite.

### 4.3. MetaCore Analysis

Metabolites with significantly different abundances (*p* < 0.05) were analyzed with MetaCore pathway, processes and pathology ontologies. Only pathways, processes and pathologies identified with a false discovery rate (FDR) less than or equal to 0.05 were accepted similar to our previous study [12].

### 4.4. Random Forest Analysis

Quantitative values of metabolites for the Control and Hemangiosarcoma groups were analyzed in R (*randomForest* Package, version 4.6-14) (Merck Research Laboratories, Kenilworth, NJ, USA) [75] with a classification algorithm to determine if these two groups were distinct based on the metabolome data. This analysis included both identified and unidentified metabolites (1123 total). 50,000 trees (*n*) were used with a *m_try_* value of 8. The quality or accuracy of the random forest model was assessed by calculation of the out of the bag (OOB) error which was 18% for this model. The F1 score for this classification model was 0.8125.

### 4.5. Statistical Analysis

Scaled data were used in the data presentation. Minimum values were imputed for missing values. Statistical significance was determined using Welch’s *t*-tests and *p* ≤ 0.05 was considered statistically significant using GraphPad Prism (San Diego, CA, USA). Results are reported as means ± SEM.

## 5. Conclusions

VC exposure is a known risk factor of hemangiosarcoma development in humans; however, the mechanism is unknown. Our study provides insight into molecules—bradykinin, FDPs, bile acids, gamma-glutamyl dipeptides and C3—that may play a role in the mechanism of hemangiosarcoma development. These molecules may be involved in other forms of sarcoma development as well. Based on our data, we suggest that measurement of taurocholate or fibrin degradation products could be clinically useful. This study also proposes new diagnostic measures for screening occupational workers at risk for developing hemangiosarcoma due to VC exposure.

## Figures and Tables

**Table 1:** Subject Demographic, Employment and Vinyl Chloride Exposure Information.

Variable	Controls	Hemangiosarcoma
Number (unless otherwise noted)	17	15
Age at sampling (years)	50.5 ± 6.3	56.2 ± 10.9
BMI (kg/m^2^)	27.1 ± 3.4 (*n* = 16)	25.3 ± 2.1 (*n* = 12)
Employment duration at sampling (years)	25.5 ± 5.7	31.9 ± 12.0
Cumulative Exposure Rank Month (CERM)	1156 ± 420.4	1266 ± 541.2
Duration of sample storage (years)	35.0 ± 5.1	27.5 ± 12.0 ^a^

^a^*p* < 0.05 vs. Controls. Group means ± standard deviations are reported.

**Table 2:** Routine Clinical Chemistries.

Analyte (Reference Range)	Controls	Hemangiosarcoma
Number (unless otherwise specified)	17	12
Albumin (3.5–5.0 mg/dL)	4.6 ± 0.2	4.4 ± 0.2
Total bilirubin (0.2–1.0 mg/dL)	0.6 ± 0.2	0.6 ± 0.2
Alkaline phosphatase (38–126 U/L)	70.5 ± 15.0	68.3 ± 36.2
AST (10–50 U/L)	24.1 ± 15.6	27.8 ± 12.1
ALT (20–70 U/L)	24.9 ± 18.4	19.8 ± 8.3
Triglycerides (10–190 mg/dL)	153 ± 49.6	201 ± 101.6 (*n* = 8)
Total cholesterol (120–200 mg/dL)	213 ± 32.6	217 ± 32.5 (*n* = 8)
Glucose (70–110 mg/dL)	101 ± 17.3	105 ± 16.1 (*n* = 8)

**Table 3:** Ontologies enriched by Hemangiosarcoma vs. Exposure.

Ontology	*p*-Value	FDR
Bile acid biosynthesis	3.45 × 10^−7^	1.17 × 10^−5^
Taurine and hypotaurine metabolism	5.74 × 10^−6^	9.76 × 10^−5^
Cholesterol metabolism	1.08 × 10^−5^	1.22 × 10^−4^
Cholesterol and bile acid homeostasis	4.56 × 10^−5^	1.10 × 10^−3^
Renal secretion of inorganic electrolytes	1.17 × 10^−4^	9.91 × 10^−4^
Renal secretion of organic electrolytes/rodent version	2.57 × 10^−4^	1.75 × 10^−3^
Regulation of lipid metabolism_Regulation of acetyl-CoA carboxylase 1 activity	3.88 × 10^−4^	2.20 × 10^−3^
Medium-chain saturated fatty acids synthesis	5.72 × 10^−4^	2.78 × 10^−3^
Upregulation of IL-8 expression in colorectal cancer	1.24 × 10^−3^	5.27 × 10^−3^
Carbohydrate metabolism_TCA and tricarboxylic acid transport	1.97 × 10^−3^	4.74 × 10^−2^
Oxidative phosphorylation	2.03 × 10^−3^	7.68 × 10^−3^
Metabolic diseases	3.28 × 10^−3^	3.93 × 10^−2^
Oxidative stress Role of Sirtuin1 and PGC1-alpha in activation of antioxidant defense system	4.31 × 10^−3^	1.46 × 10^−2^

**Table 4:** Ontologies diminished by Hemangiosarcoma vs. Exposure.

Ontology	*p*-Value	FDR
Nicotine signaling in cholinergic neurons	1.65 × 10^−5^	4.94 × 10^−4^
L-Lysine metabolism	1.06 × 10^−4^	1.20 × 10^−3^
L-Tryptophan metabolism (part 2)	1.20 × 10^−4^	1.20 × 10^−3^
Nicotine action	1.48 × 10^−4^	5.02 × 10^−3^
(L)-carnitine pathway	3.12 × 10^−4^	3.12 × 10^−3^
N-Acylethanolamines, HSRL5-transacylation pathway	3.44 × 10^−4^	2.58 × 10^−3^
Development_Neuromuscular junction	1.25 × 10^−3^	3.76 × 10^−3^
Neurophysiological process_Transmission of nerve impulse	2.60 × 10^−3^	3.89 × 10^−3^
Renal secretion of organic electrolytes/Rodent version	2.79 × 10^−3^	1.67 × 10^−2^
Cholesterol biosynthesis	4.93 × 10^−3^	2.46 × 10^−2^
Steroid metabolism_Cholesterol biosynthesis	9.27 × 10^−3^	4.64 × 10^−2^
Nicotine signaling in cholinergic neurons	1.65 × 10^−5^	4.94 × 10^−4^

## Data Availability

All the metabolome data can be found in Supplemental Materials.

## Supplementary Materials

The following are available online at https://www.mdpi.com/article/10.3390/ijms22105093/s1, Excel File S1: Metabolomic data.

## Abbreviations

ASL, angiosarcoma of the liver; B1, bradykinin receptor 1; B2, bradykinin receptor 2; C3, complement component 3; DHA, docosahexaenoic acid; DHEA, dehydroepiandrosterone; EPA, eicosapentaenoic acid; FDPs, fibrin degradation products; GC/MS, gas chromatography-mass spectrometry; GGT, gamma-glutamyl transferase; LC/MS^2^, liquid chromatography-tandem mass spectrometry; MOA, mode of action; PA, plasminogen activator; PPM, parts per million; PVC, polyvinyl chloride; TASH, toxicant associated steatohepatitis; VC, vinyl chloride; VCM, vinyl chloride monomer.
